# Health outcomes following caesarean birth- An observational study of twins discordant for mode of delivery

**DOI:** 10.1016/j.eurox.2026.100476

**Published:** 2026-07-11

**Authors:** Amanda Sturm, Indra Wäringer, Anna-Karin Wikström, Susanne Hesselman

**Affiliations:** a Department of Women’s and Children’s Health, Uppsala University, Uppsala, Sweden; b School of Medical Sciences, Örebro University, Örebro, Sweden; c Centre for Clinical Research Dalarna, Falun, Sweden

**Keywords:** Caesarean section, Multiple birth, Discordant mode of birth, Cardiovascular diseases, Metabolic diseases, Immune system diseases

## Abstract

**Introduction:**

Observational studies indicate that children born by caesarean section (CS), have an increased risk of long-term adverse health outcomes such as allergy, obesity and autoimmune disorders. A small proportion of twin pregnancies have discordant mode of delivery, where the first twin is delivered vaginally and the second twin by CS. This provides a unique opportunity to study long-term health outcomes according to mode of birth. The aim of this study was to investigate if birth by CS is associated with cardiovascular-, metabolic-, autoimmune- or allergic disorders, by using a natural experiment with combined twin birth.

**Methods:**

Pairs of twins with discordant mode of delivery between 1973 and 2020 were identified by the Swedish Medical Birth Register. Adverse long-term outcomes were captured by diagnosis and medical prescriptions identified in national registers. Associations between mode of delivery and adverse outcomes were analyzed with logistic regression and reported as odds ratios (ORs) with 95% confidence intervals (CIs). Vaginal birth (first twin) was defined as the reference group.

**Results:**

3196 twins were included. Most discordant twin births occurred from 1990 and onward (86.5%). There was no association between mode of delivery and cardiovascular- (OR: 0.88, 95% CI 0.63–1.23), metabolic- (OR 1.06, 95% CI 0.74–1.52) or allergic disorders (OR 0.99, 95% CI 0.86–1.14). Autoimmune disorders were more common after CS than vaginal delivery (4.3% vs 2.5%), OR 1.72 (95% CI 1.15–2.55).

**Conclusion:**

In twins with discordant birth modes, CS was associated with an increased likelihood of autoimmune disorders later in life, compared with vaginal birth.

## Highlights

• Discordant mode of birth in twins provides a natural experiment in studying future health.
• No association between mode of birth and cardiovascular- metabolic- or allergic disorders.
• CS increased the likelihood of autoimmune disorders compared with vaginal birth.

## Introduction

Caesarean section (CS) is one of the most commonly performed major surgical procedures worldwide [1], [2], [3]. The global rates of CS has increased over the past decades from approximately 7% in 1990–21% in 2023 [4]. Although CS can be a life-saving intervention for both mother and child [1], [3], [5], [6], it is also associated with short- and long-term health consequences. Epidemiological studies indicate that children born by CS are at increased risk of developing asthma [3], [5], [6], [7], [8], [9], [10], [11], allergy [5], [6], [9], obesity [3], [5], [6], [7], [12] and inflammatory bowel disease [10], [13], [14] in childhood or adolescence.

The evidence regarding other autoimmune disorders, including type 1 diabetes and celiac disease, is less consistent. A meta-analysis published in 2008 comprising twenty observational studies, reported a 20% increased risk of type 1 diabetes among individuals born by CS [15]. However, this association has not been confirmed in more recent population-based studies from Sweden [16] and Denmark [10]. Similarly, although several observational studies have found an increased risk of celiac disease following birth by CS [14], [17], these findings where not supported by a meta-analysis including 11 articles [18]. However, an enhanced risk of celiac disease following elective CS cannot be excluded [17]. Most studies investigate outcomes in childhood and adolescence.

Three main theories with diverse biological mechanisms have been suggested as plausible explanation for mode of delivery and short- and long-term health outcomes [19]. The first theory is lack of adequate vaginally transferred maternal microbiome during CS and its effects on the composition of the child’s microbiota and subsequent immune development [5]. Reduced exposure could have a significant impact on the newborn´s gut microbiota during the first weeks after birth [20] until several years after the procedure [21]. Alterations in the microbiota might affect the maturation and differentiation of the neonatal immune system, thereby increasing the risk of cardiovascular-, metabolic- and autoimmune diseases [5]. The second theory relates to the reduced physiological stimuli during caesarean birth which may result in lower production and release of stress hormones, potentially influencing the neurogenesis, the development of the hypothalamic-pituitary-adrenal axis and maturation of the lung and immune system [5]. The third theory suggests that epigenetic modifications induced by exposure of medications, such as synthetic oxytocin and antibiotics, or specific interventions during vaginal birth and caesarean section [22], may contribute to differences in health outcomes.

Approximately 5–10% of twin pregnancies result in discordant mode of birth [23], [24], in which the first twin is delivered vaginally and the second twin by CS. This provides a unique opportunity to study health outcomes according to mode of delivery. The aim of this study was to investigate if birth by CS is associated with cardiovascular-, metabolic-, autoimmune- or allergic disorders, by using a natural experiment with combined twin birth.

## Methods

**Fig. 1:**
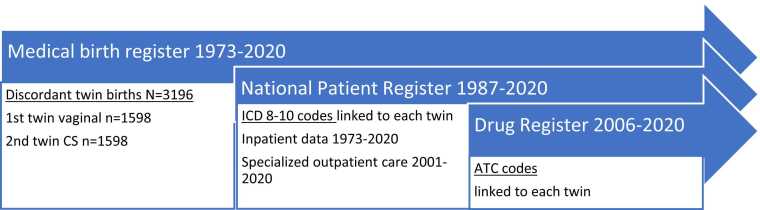
Timeline of available register data from Swedish Medical Birth Register (MBR), National Patient Register (NPR) and the National Prescription Drug Register.

This retrospective register-based cohort study included twin pairs with discordant mode of birth, first twin vaginally delivered and the second twin with CS, between the years of 1973–2020 in Sweden. Twin pairs were identified through the Swedish Medical Birth Register (MBR). Future medical diagnosis linked to each twin for cardiovascular-, metabolic, autoimmune- and allergic disorders until year 2020 were obtained from the National Patient Register (NPR), a register containing information on specialized in- and outpatient care with national coverage since 1987 and 2001 respectively. Medical prescriptions was retrieved from the Swedish National Prescription Drug Register from 2006 until year 2020 and linked to each twin (Fig. 1). Data were provided by the National Board of Health and Welfare and Statistics Sweden.

The following information was provided from MBR: decade of birth, maternal age (years at delivery), maternal body mass index (BMI) (kg/m2) registered at first trimester, mothers’ country of birth (divided into Nordic or non-Nordic origin), living situation (dichotomized into cohabitation/non-cohabitation), smoking and snuff use 3 months before pregnancy and during early pregnancy, parity (nulliparous/parous), previous birth by CS, assisted reproduction (including assisted fertilization, ovulation induction, intracytoplasmic sperm injection and other infertility treatments). Presence of pregestational disorders (diabetes mellitus, chronic kidney disease, epilepsy, systemic lupus erythematosus (SLE), chronic hypertension, asthma and lung disease) were based on checkboxes in standardized templates in MBR, and gestational disorders including preterm prelabour rupture of membranes (PPROM) and gestational hypertensive disorders (pregnancy induced hypertension and preeclampsia) from diagnostic codes. Birth records in MBR further provided information of gestational length (days), operative vaginal birth or attempt (vacuum extraction or forceps), fetal presentation (divided into cephalic, breech or other), sex (male/female), birthweight (grams), Apgar score at 5 min (analysed for liveborn infants and presented as proportion with scores <7 at 5 min of age), perinatal infection, respiratory distress, congenital malformation and perinatal death (defined as stillbirth, intrapartum death and neonatal death during the first 27 days of life). Details of source of variables and coverage is provided in Supplementary A.

### Exposure

Mode of delivery for each twin: vaginal (including instrumental) or CS, was entered in checkboxes in medical charts and forwarded to MBR.

### Outcomes

Health outcomes in child- or adulthood was based on International Statistic Classification of Diseases and Related Health Problems (ICD) codes in NPR and Anatomical Therapeutic Chemical (ATC) codes from the National Prescription Drug Register. Cardiovascular disorders included diagnoses for hypertensive diseases, stroke and ischemic heart diseases and prescription of antihypertensive drugs. Metabolic disorders included the diagnosis of type 2 diabetes or diabetes medication (insulins and analogues, blood glucose lowering drugs and other drugs used in diabetes), hypothyroidism and thyroid preparations, antithyroid preparations and iodine therapy, polycystic ovary syndrome, obesity, prescription of lipid modifying agents, and prescription of anti-obesity preparations (excluding diet products). Autoimmune disorders included diagnostic codes for type 1 diabetes, celiac disease, systemic connective tissue disorders, juvenile arthritis and inflammatory polyarthritis or prescriptions of immunosuppressants and corticosteroids for systemic use. Allergic disorders consisted of diagnoses for asthma, urticaria and erythema and prescription of drugs for asthma and allergy (including drugs for obstructive airway diseases and antihistamine preparations for systemic use) (Supplementary A). Health outcomes were analysed after excluding perinatal deaths (n = 59).

### Statistics

The incidence of twin births in Sweden 1973–2020 was collected from open-source data from the National Board of Health and Welfare (https://www.socialstyrelsen.se/statistik-och-data/statistik/statistikdatabasen/). The proportion of twin births with discordant mode of delivery was calculated per decade. Statistical analyses were performed using SPSS 28.0 and Jamovi (version 2.6), an open-source statistical software (The Jamovi project, 2025, https://www.jamovi.org/). Descriptive analysis was used to calculate numbers and percentage of prevalence, mean values and standard deviations (SD). Comparative analysis of categorical data was done using Chi-square test and continuous data by using the Student’s *t*-test. A p-value of < 0.05 was considered significant. Associations between mode of delivery and long-term health outcomes were analysed with logistic regression and reported as odds ratios (ORs) with 95% confidence intervals (CIs). Vaginal birth (twin 1) was the reference group. Directed acyclic graph (DAG) was constructed to suggest adjustments to estimate the total effect of CS on the outcomes. The model included Apgar score < 7 at birth, birthweight and operative vaginal birth or attempt (Supplementary B).

### Ethical considerations

Ethical application was approved on January 28, 2020, by the Swedish Ethical Review Authority; Diary number 2019–04925, with amendment March 4th, 2022, referenced as 2022–00922–02. The data used was pseudonymised and stored on a secure storage surface. Consent from participants to be included in the study was not required as Swedish legislation exempts central government registers from the standard requirement to obtain informed consent. Access to the raw data is restricted to protect patient privacy and comply with ethical guidelines.

## Results

**Fig. 2:**
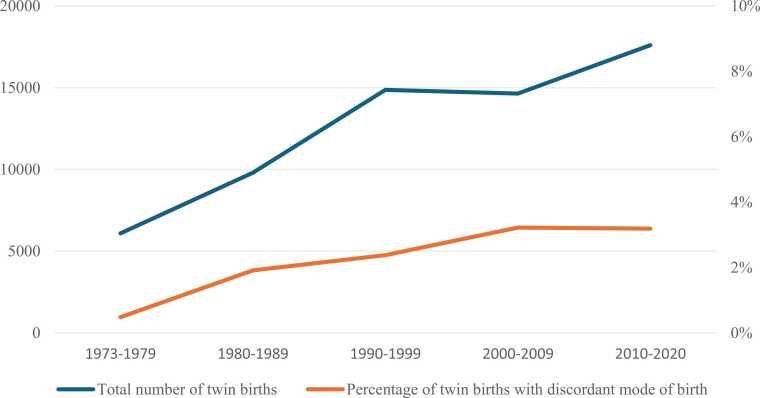
Total number of twin births and percentage of twin births with discordant mode of birth in Sweden between 1973 and 2020.

A total of 3196 twins with discordant mode of birth were identified between 1973 and 2020. Both the number of multifetal births in Sweden and the proportion of twins with discordant mode of birth increased during the study period, with the latter rising from 0.48% to 3.19% (Fig. 2).

Most discordant births occurred from 1990 and onwards (86.5%). The mean maternal age at delivery was 30.9 years. Smoking was reported by 12.1% in early pregnancy and 104/1598 (6.5%) had a pregestational medical disorder (Table 1).

**Table 1:** Characteristics of the mothers with discordant mode of birth of twins 1973–2020 (n = 1598).

	Not reported n (%) =	Rate or mean n (%) =
**Decade of birth,** n (%) =		
1973–79	-	29 (1.8)
1980–89	-	187 (11.7)
1990–99	-	350 (21.9)
2000–09	-	471 (29.5)
2010–20	-	561 (35.19)
**Maternal age** (years), mean [SD]	-	30.9 [5.14]
≥ 35, n (%)	-	399 (25.0)
**BMI** (kg/m^2^), mean [SD]	367 (23.0)	24.9 [4.60]
≥ 30, n (%)		168 (13.6)
**Nordic country of birth**^a^**,** n (%)	5 (0.3)	1329 (83.4)
**Cohabitation,** n (%)	26 (1.6)	1371 (87.2)
**Smoking,** n (%)		
Prior pregnancy	613 (38.4)	147 (14.9)
Early pregnancy	171 (10.7)	173 (12.1)
**Snuff use,** n (%)		
Prior pregnancy	602 (37.7)	32 (3.2)
Early pregnancy	588 (36.8)	10 (1.0)
**Parity,** n (%)	-	
Nulliparous	-	584 (36.5)
Parous	-	1014 (63.5)
**Prior CS,** n (%)	82 (5.1)	68 (4.5)
**Pregestational disorder**^b^**,** n (%)	-	104 (6.5)
**PPROM,** n (%)	-	26 (1.6)
**Hypertensive disorder**^c^, n (%)	-	157 (9.8)
**Assisted reproduction,** n (%)	412 (26)	217 (13.6)

SD=standard deviation; BMI = body mass index; PPROM = preterm prelabour rupture of membranes; CS = caesarean section.

^a^Maternal country of birth.

^b^Diabetes mellitus, chronic kidney disease, epilepsy, systemic lupus erythematosus (SLE), chronic hypertension, asthma and lung disease.

^c^Pregnancy induced hypertension and preeclampsia.

Out of 3196 included infants, 37.1% were born preterm (< 37 weeks). Successful operative vaginal birth or an attempt prior to CS was more common in the first than the second twin (p < 0.001). Fetal distress was more common for the second twin with an Apgar score < 7 at 5 min reported in 39 (2.5%) of first and in 234 (15.1%) of second liveborn twins (p < 0.001). Respiratory distress was also more common among the second born twin than the first twin, but rates of perinatal infections were equal (Table 2).

**Table 2:** Characteristics of first (vaginal) and second (CS) born twin at birth (n = 3196).

		Twin 1 (vaginal) n = 1598	Twin 2 (CS) n = 1598	p-value*
**Gestational length** (days) *Missing n = 4*	mean [SD]	257.96	258.04	
	< 37 weeks	592 (37.1)	591 (37.1)	0.992
**Operative vaginal birth** (or attempt)		209 (13.1)	145 (9.1)	< 0.001
**Fetal presentation** *Missing n = 657*				< 0.001
	Cephalic	1319 (97.1)	347 (29.4)	
	Breech	31 (2.3)	448 (38.0)	
	Other	9 (0.7)	385 (32.6)	
**Sex**	Male	812 (50.8)	902 (56.4)	< 0.001
	Female	786 (49.2)	696 (43.6)	
**Birthweight** (grams) *Missing n = 21*	mean [SD]	2603 [601]	2609 [596]	0.776
**Apgar score**^a^ < 7 at 5 min *Missing n = 80*		39 (2.5)	234 (15.1)	< 0.001
**Perinatal infection**		33 (53.2)	29 (46.8)	0.608
**Respiratory distress**		90 (36.7)	155 (63.3)	< 0.001
**Congenital malformation**		75 (4.7)	73 (4.6)	0.866
**Perinatal death**^**b**^		25 (1.6)	34 (2.1)	0.237

Data presented as numbers (n = ) and frequencies (%) or means with standard deviations.

CS = caesarean section.

^a^Liveborn (n = 3183); ^b^stillbirth (n = 10), intrapartum death (n = 3) and neonatal death first 27 days (n = 46).

*Chi-square test or Student`s *t*-test.

Mean age follow up was 16.9 years (SD 11.0), range 0–47. There was no crude association between birth by CS and cardiovascular- (OR 0.88, 95% CI 0.63–1.23), metabolic- (OR 1.06, 95% CI 0.74–1.52) or allergic disorders (OR 0.99, 95% CI 0.86–1.14). Autoimmune disorders were recorded in 107 out of 3137 (3.4%) twins and were more commonly reported in the second than the first twin (4.3% vs 2.5%), OR 1.72 (95% CI 1.15–2.55). After adjusting for Apgar score, birthweight and operative vaginal birth or attempt, the association between CS and autoimmune disorders remained (aOR 1.89, 95% CI 1.26–2.84) (Table 3).

**Table 3:** Long-term adverse health outcomes of first (vaginal) and second (CS) born twin (n = 3137).

	Total n (%)	Twin 1 (vaginal)	Twin 2 (CS)	OR (95% CI)	aOR* (95% CI)
		n (%) =	n (%) =		
**Cardiovascular disorders**^a^	143 (4.6)	76 (4.8)	67 (4.3)	0.88 (0.63–1.23)	0.91 (0.64–1.29)
**Metabolic disorders**^b^	121 (3.9)	59 (3.8)	62 (4.0)	1.06 (0.74–1.52)	1.13 (0.77–1.65)
**Autoimmune disorders**^c^	107 (3.4)	40 (2.5)	67 (4.3)	1.72 (1.15–2.55)	1.89 (1.26–2.84)
**Allergic disorders**^d^	1772 (56.5)	891 (56.6)	881 (56.3)	0.99 (0.86–1.14)	0.99 (0.85–1.14)

CS = caesarean section; OR, aOR = odds ratio, adjusted odds ratio; CI = confidence interval.

^a^Hypertension, antihypertensives, ischemic heart diseases and stroke.

^b^Diabetes mellitus type 2, diabetes medication, polycystic ovary syndrome (PCOS), hypothyroidism, thyroid therapy, obesity, antiobesity preparations and lipid modifying agents.

^c^Diabetes mellitus type 1, celiac disease, juvenile arthritis, inflammatory polyarthritis, systemic connective tissue disorders, immunosuppressants and corticosteroids for systemic use.

^d^Asthma drugs, allergy drugs, asthma, urticaria and erythema.

*Adjusted for Apgar, birthweight and operative vaginal birth (n = 3057).

## Discussion

In twins with discordant mode of birth, delivery by emergency CS of the second twin was associated with autoimmune disorders compared with vaginal birth of the first twin. This association remained after adjusting for birth related factors. There was no association between mode of delivery and offspring’s future cardiovascular-, metabolic- or allergic disorders.

The observed association between CS and autoimmune disorders was stronger, but in accordance, with a previous meta-analysis which demonstrated a 20% increased risk of type 1 diabetes after CS compared with vaginal birth [15]. In contrast, large population-based studies from the Nordic countries have not confirmed an increased risk of diabetes among offspring born by CS [10], [16]. Furthermore, a meta-analysis including 11 articles found no evidence of an association between CS and celiac disease [18].

Both genetic and environmental factors are risk factors for autoimmune disorders, that could be minimised by studying twins. Significant differences in the incidence of birth asphyxia and respiratory distress between the first and second born twin was observed. An increased risk of fetal distress for the second twin compared with the first twin is well established, and especially in case of combined mode [25], [23]. To our knowledge there is no association between fetal asphyxia and the development of autoimmune diseases, and our results remained after adjusting for Apgar score.

Contrary to findings from previous epidemiological studies, we found no association between mode of birth and allergic or metabolic disorders. A large meta-analysis comprising 23 studies, reported a 20% increased risk of childhood asthma if born by CS [8], and a 23% increased odds for allergic rhinitis [9], compared with vaginal birth. Furthermore, a meta-analysis of 15 studies demonstrated a strong association between CS and subsequent development of overweight and obesity in offspring [26]. However, these associations might be influenced or caused by underlying genetic-, pregnancy- and environmental factors. The discrepancies between our findings and prior research may also result from detection bias due to the sibling-design. More than half of offspring’s in our cohort, irrespective of mode of birth, had allergic disorders, compared with approximately one third in the general Swedish population [27]. In addition, one third was born preterm, reflecting a potential elevated baseline risk of asthma, metabolic disorders and cardiovascular diseases later in life. Nevertheless, a recent expert review concluded that CS at term remains associated with an increased risk of asthma, inflammatory bowel disease and overweight compared with vaginal birth [28]. It is also worth to note that prior literature of consequences of CS have focused on health outcomes in childhood and adolescence. In our study mean age for follow up was 16.9 years but ranged from 0 to 47 years.

There are differences in microbiome transmission and intrapartum exposure to stress between elective CS and nonelective CS [29]. Levels of cytokines is affected by whether the woman has been in active phase of labour before the procedure is performed [30]. A case-control study found a higher likelihood of celiac disease after elective CS, but not with emergency CS [17]. In our cohort, both twins have been exposed to active labour, stress and potentially, the second twin have also been exposed to the mother´s vaginal microbiome before the CS. A survey of 257 pairs of twins revealed no association with time ruptured membranes and asthma or allergy [31]. In our cohort, only a small proportion had PPROM (1.6%). We adjusted for operative vaginal birth or attempt but had no information about manual interventions on the second twin and fetal station at CS.

### Strengths and limitations

The main strength of our study lies in the innovative study design which can be seen as a natural experiment. Although we could not control for zygosity, the comparison between two groups exposed to the same genetic-, intrauterine- and social factors reduced confounding. Another strength is the use of registers, with linked information between specific birth data and future medical diagnosis at a population level, restricting selection bias and omitting recall bias. Both the MBR, including 98% of all births in Sweden, and the Swedish National Inpatient Register, a part of NPR, are considered reliable containing high quality and valid data [32]. Since most discordant twin births occurred from 1990 and onward (86.5%) the time to follow-up was likely being too short for several outcomes, e.g., cardiovascular disorders. This restricts interpretation and validation of long-term medical consequences of birth by CS. However, both the first and second twin had the same time at risk and time to follow-up. Due to restricted numbers of specific outcomes, we investigated groups of medical disorders and not individual diagnoses. Categorization of conditions was based on similarity between conditions and decided prior to analysis. Register based data and categorization of disorders make the resolution of results poor, as diagnosis- and prescription codes used are broad and do not provide any measure of the severity of illness. Diagnostic codes were obtained from NPR, a register with a higher proportion of valid diagnoses for severe diseases in specialized care but not reported from primary care [33]. This might result in an underestimation of the prevalence of mild or disorders treated in primary care. For this purpose, additional information for prescriptions of medications from the National Prescription Drug Register reduced this limitation.

## Conclusion

By using a natural experiment investigating future health in twins with discordant mode of birth, CS increased the likelihood of autoimmune disorders compared with vaginal birth. This strengthens a causal relationship of mode of birth and offsprings health. Further research on biological pathways and strategies to mitigate the risk, for example transferred vaginal microbiome at CS, are warranted.

## CRediT authorship contribution statement

**Indra Wäringer:** Writing – original draft. **Anna-Karin Wikström:** Writing – review & editing. **Amanda Sturm:** Writing – review & editing, Supervision. **Susanne Hesselman:** Writing – review & editing, Visualization, Supervision, Formal analysis, Conceptualization.

## Authors contribution

SH designed the study, planned and arranged the dataset with technical support of PW, and conducted the analysis. IW wrote the first draft with technical and intellectual support of SH and AS. AS finalized the manuscript. All authors approved the final version.

## Funding

SH was supported by Centre for clinical research Dalarna (grant CKFUU-101147) and ALF-funding Uppsala. AS was supported by Ester Åsberg Lindberg foundation.

## Declaration of Competing Interest

The authors declare the following financial interests/personal relationships which may be considered as potential competing interests: Amanda Sturm reports financial support was provided by Ester Åsberg Lindberg foundation. Susanne Hesselman reports financial support was provided by Centre for clinical research Dalarna. Co-author Susanne Hesselman is a EJOG editorial board member. Given her role as editorial board member, S.H had no involvement in the peer review of this article and had no access to information regarding its peer review. Full responsibility for the editorial process for this article was delegated to another journal editor. If there are other authors, they declare that they have no known competing financial interests or personal relationships that could have appeared to influence the work reported in this paper.
