# Supplementary material for: Health outcomes following caesarean birth- An observational study of twins discordant for mode of delivery
Source: Eur J Obstet Gynecol Reprod Biol X. 2026 Jul 11;31:100476. doi: 10.1016/j.eurox.2026.100476 (PMC13396910; doi:10.1016/j.eurox.2026.100476)
Supplement: Supplementary file 1 — Supplementary material [file mmc1.docx]

Supplementary A – Details regarding covariates and health outcomes.

| **Name of the variable** | **Source** | **Note** | **Data available since (year)** |
| --- | --- | --- | --- |
| Decade of delivery | MBR^a^ | - | 1973- |
| Age | MBR | - | 1973- |
| BMI | MBR | Calculated based on weight and height data | 1982-1989, 1992-^*^ |
| Smoking | MBR | Self-reported data. Transformed to a binary scale (yes or no) | 1982- |
| Snuff use | MBR | Self-reported data | 1999- |
| Cohabitation | MBR | Transformed to a binary scale (yes or no) | 1973- |
| Nordic country of birth | MBR | Transformed to a binary scale (yes or no) | 1973- |
| Pregestational disorder | MBR | Self-reported data. Includes diabetes mellitus, chronic kidney disease, epilepsy, systemic lupus erythematosus (SLE), chronic hypertension, asthma and lung disease | 1973- |
| Parity | MBR | Dichotomized into nulliparous or parous | 1973- |
| Prior CS | MBR | - | 1973- |
| Assisted  Reproduction | MBR | Includes assisted fertilization, ovulation induction, intracytoplasmic sperm injection (ICSI) and other treatment in case of involuntary infertility | 1995- |
| Gestational length | MBR | - | 1973- |
| Operative vaginal birth (or attempt) | MBR | Includes vacuum extraction and forceps | 1973- |
| Fetal presentation | MBR | Divided into cephalic, breech or other | 1982 |
| Sex | MBR | - | 1973- |
| Birthweight | MBR | - | 1973- |
| Height | MBR | - | 1973- |
| Apgar score | MBR | - | 1973- |
| Congenital malformation | MBR | - | 1973- |
| Perinatal death | MBR | Includes stillbirth, intrapartum death and neonatal death during the first 27 days of life | 1973- |
| Hypertensive disorder of pregnancy | MBR | ICD-10: O10-O11, O13-O15  ICD-9: 642A-642H, 642X  ICD-8: 401-405, 637 | 1973- |
| PPROM | MBR | ICD-10: O42  ICD-9: 658B  ICD-8: 634,95 | 1973- |
| **Cardiovascular disorders** |  |  |  |
| Hypertension | NPR | ICD-10: I10-I13, I15  ICD-9: 401-405  ICD-8: 400-404 | 1973- |
| Antihypertensives | LMED | ATC: C02-C03, C07-C09 | 2005- |
| Ischemic heart diseases | NPR | ICD-10: I20-I25  ICD-9: 410-414  ICD-8: 410-414 | 1973- |
| Stroke | NPR | ICD-10: 161-I63  ICD-9: 431, 434X  ICD-8: 431, 433-438 | 1973- |
| **Metabolic disorders** |  |  |  |
| Diabetes mellitus type 2 | NPR | ICD-10: E11  ICD-9: 250  ICD-8: 250 | 1973- |
| Diabetes medication | LMED | ATC: A10A, A10B, A10X | 2005- |
| Polycystic ovary syndrome (PCOS) | NPR | ICD-10: E282  ICD-9: 256E  ICD-8: 25690 | 1973- |
| Hypothyroidism | NPR | ICD-10: E03  ICD-9: 243  ICD-8: 244 | 1973- |
| Thyroid therapy | LMED | ATC: H03 | 2005- |
| Obesity | NPR | ICD-10: E66 | 1997- |
| Antiobesity preparations | LMED | ATC: A08 | 2005- |
| Lipid modifying agents | LMED | ATC: C10 | 2005- |
| **Autoimmune disorders** |  |  |  |
| Diabetes mellitus type 1 | NPR | ICD-10: E10  ICD-9: 250  ICD-8: 250 | 1973- |
| Celiac disease | NPR | ICD-10: K900 | 1997- |
| Juvenile arthritis | NPR | ICD-10: M08-M09 | 1997- |
| Inflammatory polyarthropathies | NPR | ICD-10: M05-M14 | 1997- |
| Systemic connective tissue disorders | NPR | ICD-10: M30-M31, M33-M36 | 1997- |
| Immunosuppressants | LMED | ATC: L04 | 2005- |
| Corticosteroids for systemic use | LMED | ATC: H02A | 2005- |
| **Allergic disorders** |  |  |  |
| Asthma drugs | LMED | ATC: R03 | 2005- |
| Allergy drugs | LMED | ATC: R06 | 2005- |
| Nasal preparations | LMED | ATC R01 | 2005- |
| Asthma | NPR | ICD-10: J45-J46 | 1997- |
| Urticaria and erythema | NPR | ICD-10: L50-L54 | 1997- |

MBR = Medical Birth Register; BMI = body mass index; CS = caesarean section; NPR = National Patient Register; ICD = International Statistic Classification of Diseases and Related Health Problems; LMED = National Prescribed Drug Register; ATC = Anatomical Therapeutic Chemical

^*^For the years 1990-1991, weight data is completely missing because the reported weights were unreasonably high (probably the effect of a journal revision during those years) and were therefore blanked.

^a^A cross in a checkbox or a diagnostic code linked to birth record reflects the presence of a condition ie there is no box for negation or “No”.
