# Supplementary material for: Health outcomes following caesarean birth- An observational study of twins discordant for mode of delivery
Source: Eur J Obstet Gynecol Reprod Biol X. 2026 Jul 11;31:100476. doi: 10.1016/j.eurox.2026.100476 (PMC13396910; doi:10.1016/j.eurox.2026.100476)

**Supplementary B.** Suggested adjustments for estimate of total effect of caesarean section (CS) on autoimmune diseases.


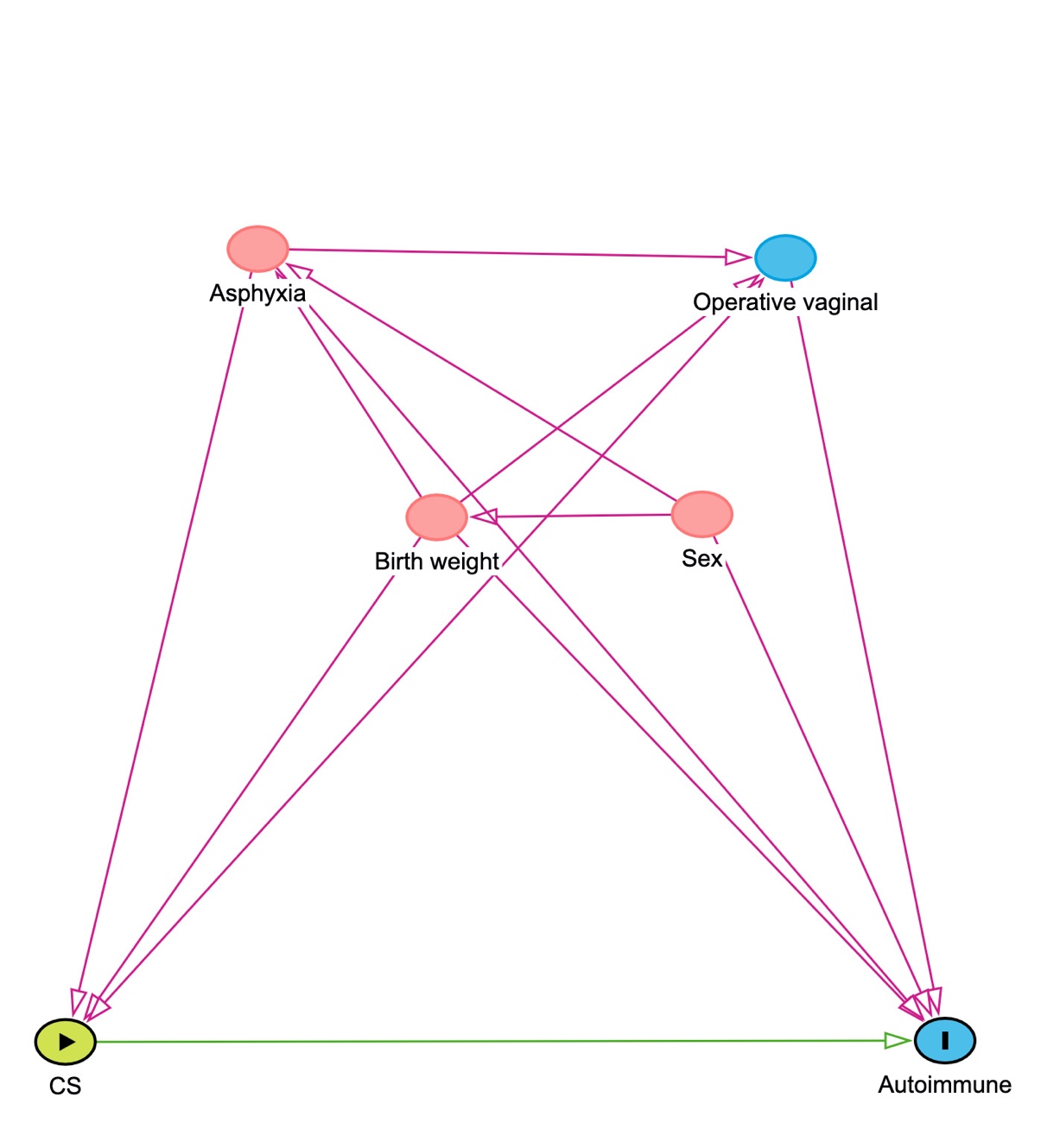

Supplement: Supplementary file 2 — Supplementary material [file mmc2.docx]
